# Editorial: Exploring SARS-CoV-2 interactions in aging and comorbid populations

**DOI:** 10.3389/fmed.2026.1831810

**Published:** 2026-04-02

**Authors:** Carmine Siniscalchi, Pierpaolo Di Micco

**Affiliations:** 1 Department of Medicine and Surgery, University of Parma, Parma, Italy; 2 Internal Medicine Department, Ospedale Santa Maria delle Grazie, Pozzuoli, Italy

**Keywords:** aging adults, comorbid populations, platelet, post COVID, SARS-CoV-2

The coronavirus disease 2019 (COVID-19) pandemic has profoundly reshaped the landscape of global health, revealing complex interactions between viral infection, aging, and chronic comorbidities. Since the early stages of the pandemic, it has become increasingly clear that older adults and individuals with multiple chronic conditions represent the populations most vulnerable to severe clinical outcomes following infection with severe acute respiratory syndrome coronavirus 2 (SARS-CoV-2). The Research Topic “*Exploring SARS-CoV-2 interactions in aging and comorbid populations*” was conceived to investigate these multifaceted relationships, bringing together contributions that explore biological mechanisms, clinical predictors, epidemiological trends, and long-term consequences of COVID-19 in frail and multimorbid populations.

Frailty has emerged as a particularly relevant determinant of outcomes in older adults with COVID-19. In this context, the Clinical Frailty Scale has been widely adopted as a practical tool for assessing vulnerability among hospitalized patients. Evidence from hospitalized cohorts has shown that higher frailty scores are strongly associated with increased mortality and poorer clinical outcomes in patients with COVID-19 pneumonia (Siniscalchi, Ticinesi et al.). Frailty reflects a reduction in physiological reserve and an impaired ability to cope with acute stressors such as severe infections. As a consequence, frail patients may experience disproportionate inflammatory responses and organ dysfunction following viral infection, leading to worse outcomes even when controlling for chronological age. Beyond COVID-19 itself, frailty and geriatric syndromes such as delirium and multimorbidity have also been shown to strongly influence in-hospital mortality among older adults admitted to internal medicine wards (1).

Another important aspect highlighted in this Research Topic concerns the role of hematological and inflammatory biomarkers in predicting disease progression. Several studies have explored the prognostic value of routine laboratory parameters in hospitalized patients with SARS-CoV-2 infection. For example, predictive models based on circulating biomarkers have been proposed to identify patients at higher risk of severe disease (Xiaoyan et al.). Hematological parameters, including leukocyte and platelet counts, may reflect the complex interplay between inflammation, immune activation, and endothelial dysfunction that characterizes severe COVID-19 [Matviichuk et al., (2)]. In particular, platelet abnormalities have attracted growing attention as potential markers of disease severity. In a large retrospective cohort of hospitalized patients with COVID-19 pneumonia, thrombocytopenia at hospital admission was independently associated with increased mortality, suggesting that platelet dynamics may represent an accessible and clinically meaningful prognostic indicator (2).

Beyond laboratory markers, biochemical and hematological alterations have also been described among individuals with serological evidence of previous SARS-CoV-2 infection (Izekenova et al.). Radiological and clinical features also play a crucial role in understanding disease severity. Chest imaging, particularly computed tomography, has been widely used to assess the extent of pulmonary involvement during the acute phase of infection. Radiological severity scores correlate closely with respiratory impairment and systemic inflammation, reflecting the degree of lung injury induced by the virus.

Sex-related biological differences represent another emerging dimension in the study of COVID-19 pathophysiology. From the earliest reports of the pandemic, epidemiological data consistently indicated that males were more likely to develop severe disease and experience higher mortality compared with females. However, the mechanisms underlying these differences remain complex and multifactorial. Analyses conducted in large hospital cohorts have shown that women may exhibit milder inflammatory responses and reduced mortality despite older age and greater frailty, suggesting intrinsic biological differences in host response to SARS-CoV-2 infection (Siniscalchi, Guerra et al.). Potential explanations include hormonal influences on immune responses, sex-related differences in the expression of viral entry receptors, and genetic factors associated with immune regulation.

The interaction between SARS-CoV-2 infection and the coagulation system has also emerged as a defining characteristic of severe COVID-19. A prothrombotic state, driven by endothelial dysfunction, inflammatory activation, and platelet hyperreactivity, contributes to the development of both macrovascular and microvascular thrombosis. Venous thromboembolism has been widely reported among hospitalized patients with COVID-19, particularly in those with severe respiratory failure. Clinical factors related to disease severity and respiratory support may further influence thrombotic risk. In this regard, observational data suggest that the use of non-invasive ventilation during hospitalization may be associated with an increased risk of venous thromboembolism, possibly reflecting the severity of respiratory compromise and the systemic inflammatory response (3).

Beyond COVID-19 infection itself, pharmacological factors may also influence thrombotic and inflammatory pathways. Experimental and clinical studies have suggested that statins may exert pleiotropic effects on endothelial function, inflammation, and coagulation pathways (4). Observational data from large registries have further suggested that statin use may be associated with improved outcomes in patients with venous thromboembolism and pulmonary embolism (5–7) highlighting the complex interplay between cardiovascular risk factors, thrombosis, and systemic inflammation in vulnerable populations.

From a broader epidemiological perspective, the determinants of severe COVID-19 outcomes vary across geographic regions and healthcare systems. A comprehensive literature review of the Asia-Pacific region highlighted multiple risk factors associated with severe disease, including advanced age, chronic comorbidities, and delayed access to healthcare (Thompson et al.). Similarly, studies conducted in intensive care settings have identified several predictors of mortality among critically ill COVID-19 patients, emphasizing the role of comorbidities, organ dysfunction, and inflammatory burden (Sathyadas et al.).

Beyond acute clinical outcomes, the pandemic has also revealed the broader social and health consequences of SARS-CoV-2 infection, particularly among older populations. Lockdowns, social isolation, and disruptions in healthcare services have significantly affected the physical and psychological wellbeing of older adults worldwide. Studies included in this Research Topic emphasize that quality of life during the pandemic is influenced not only by biological vulnerability but also by socioeconomic and demographic factors. For example, population-based analyses have shown that gender differences and social determinants significantly influenced changes in daily routines and health-related quality of life among older adults following the pandemic (Cameselle-Lago et al.).

In conclusion, the Research Topic “*Exploring SARS-CoV-2 interactions in aging and comorbid populations*” provides valuable insights into the biological, clinical, and social determinants of COVID-19 outcomes in older adults and individuals with chronic diseases.

## References

[1] Siniscalchi C Di Micco P Guerra A Nouvenne A Cerundolo N Parise A . Clinical and geriatric predictors of in-hospital mortality in older adults admitted to internal medicine wards: a retrospective observational study. J Clin Med. (2025) 14:6726. doi: 10.3390/jcm14196726 41095806 PMC12525266

[2] Siniscalchi C Di Micco P Guerra A Simoni R Magro J Parise A . Platelet count and clinical outcomes in hospitalized patients with COVID-19 pneumonia. Front Med. (2025) 12:1614447. doi: 10.3389/fmed.2025.1614447 41140677 PMC12549574

[3] Siniscalchi C Ticinesi A Nouvenne A Guerra A Parise A Finardi L . Non-invasive ventilation support during hospitalization for SARS-CoV-2 and the risk of venous thromboembolism. J Clin Med. (2024) 13:2737. doi: 10.3390/jcm13102737 38792278 PMC11122199

[4] Siniscalchi C Basaglia M Riva M Meschi M Meschi T Castaldo G . Statins effects on blood clotting: a review. Cells. (2023) 12:2719. doi: 10.3390/cells12232719 38067146 PMC10706238

[5] Siniscalchi C Muriel A Suriñach Caralt JM Bikdeli B Jiménez D Lobo JL . Statin use and 30-day mortality in patients with acute symptomatic pulmonary embolism. J Thromb Haemost. (2022) 20:1839–51. doi: 10.1111/jth.15753 35510755

[6] Siniscalchi C Bikdeli B Jiménez D Suriñach JM Demelo-Rodríguez P Moustafa F . Statin use and mortality in patients with deep vein thrombosis. Data from the RIETE Registry. Thromb Res. (2024) 236:88–96. doi: 10.1016/j.thromres.2024.02.024 38417300

[7] Siniscalchi C Quintavalla R Rocci A Riera-Mestre A Trujillo-Santos J Suriñach JM . Statin and all-cause mortality in patients receiving anticoagulant therapy for venous thromboembolism. Data from the RIETE registry. Eur J Intern Med. (2019) 68:30–5. doi: 10.1016/j.ejim.2019.07.028 31427187

